# *Bordetella* Adenylate Cyclase Toxin Mobilizes Its β_2_ Integrin Receptor into Lipid Rafts to Accomplish Translocation across Target Cell Membrane in Two Steps

**DOI:** 10.1371/journal.ppat.1000901

**Published:** 2010-05-13

**Authors:** Ladislav Bumba, Jiri Masin, Radovan Fiser, Peter Sebo

**Affiliations:** 1 Institute of Microbiology AS CR v.v.i., Prague, Czech Republic; 2 Faculty of Science, Charles University, Prague, Czech Republic; 3 Institute of Biotechnology AS CR v.v.i, Prague, Czech Republic; University of California Los Angeles, United States of America

## Abstract

*Bordetella* adenylate cyclase toxin (CyaA) binds the α_M_β_2_ integrin (CD11b/CD18, Mac-1, or CR3) of myeloid phagocytes and delivers into their cytosol an adenylate cyclase (AC) enzyme that converts ATP into the key signaling molecule cAMP. We show that penetration of the AC domain across cell membrane proceeds in two steps. It starts by membrane insertion of a toxin ‘translocation intermediate’, which can be ‘locked’ in the membrane by the 3D1 antibody blocking AC domain translocation. Insertion of the ‘intermediate’ permeabilizes cells for influx of extracellular calcium ions and thus activates calpain-mediated cleavage of the talin tether. Recruitment of the integrin-CyaA complex into lipid rafts follows and the cholesterol-rich lipid environment promotes translocation of the AC domain across cell membrane. AC translocation into cells was inhibited upon raft disruption by cholesterol depletion, or when CyaA mobilization into rafts was blocked by inhibition of talin processing. Furthermore, CyaA mutants unable to mobilize calcium into cells failed to relocate into lipid rafts, and failed to translocate the AC domain across cell membrane, unless rescued by Ca^2+^ influx promoted *in trans* by ionomycin or another CyaA protein. Hence, by mobilizing calcium ions into phagocytes, the ‘translocation intermediate’ promotes toxin piggybacking on integrin into lipid rafts and enables AC enzyme delivery into host cytosol.

## Author Summary

The adenylate cyclase toxin (CyaA) of pathogenic *Bordetellae* eliminates the first line of host innate immune defense. It penetrates myeloid phagocytes, such as neutrophils, macrophage or dendritic cells, and subverts their signaling by catalyzing an extremely rapid conversion of intracellular ATP to the key signaling molecule cAMP. This efficiently inhibits the oxidative burst and complement-mediated opsonophagocytic killing of bacteria, thus enabling the pathogen to colonize host airways. We show that translocation of CyaA into phagocyte cytosol occurs in two steps. The toxin first binds the integrin CD11b/CD18 and inserts into phagocyte membrane to mediate influx of calcium ions into cells. This promotes relocation of the toxin-receptor complex into specific lipid microdomains within cell membrane called rafts. The increased concentrations of cholesterol within rafts and their particular lipid organization then support translocation of the adenylate cyclase enzyme directly into the cytoplasmic compartment of cells. The mechanism of CyaA penetration into cells sets a new paradigm for membrane translocation of toxins of the RTX family.

## Introduction

The secreted adenylate cyclase toxin-hemolysin (CyaA, ACT, or AC-Hly) plays a key role in virulence of *Bordetellae*. This multifuctional protein binds the α_M_β_2_ integrin (CD11b/CD18, CR3 or Mac-1) of myeloid phagocytic cells and delivers into their cytosol a calmodulin-activated adenylate cyclase enzyme that ablates bactericidal capacities of phagocytes by uncontrolled conversion of cytosolic ATP to the key signaling molecule cAMP [1]–[5]. In parallel, the hemolysin moiety of CyaA forms oligomeric pores that permeabilize cell membrane for monovalent cations and contribute to overall cytoxicity of CyaA towards phagocytes [6]–[10].

The toxin is a 1706 residues-long protein, in which a calmodulin-activated adenylate cyclase (AC) enzyme domain of ∼400 N-terminal residues is fused to a ∼1300 residue-long RTX (Repeats in ToXin) cytolysin moiety [11]. The latter consists itself of three functional domains typical for RTX hemolysins. It harbors, respectively, (i) a hydrophobic pore-forming domain, (ii) a segment recognized by the protein acyltransferase CyaC, activating proCyaA by covalent post-translational palmitoylation at ε-amino groups of Lys_860_ and Lys_983_ [12], [13], and (iii) an assembly of five blocks of the characteristic glycine and aspartate-rich nonapeptide RTX repeats that form numerous (∼40) calcium-binding sites [14].

Since no structural information on the RTX cytolysin moiety is available, the mechanistic details of toxin translocation across the lipid bilayer of cell membrane remain poorly understood. Delivery of the AC domain into cells occurs directly across the cytoplasmic membrane, without the need for toxin endocytosis [15] and requires structural integrity of the CyaA molecule [6], unfolding of the AC domain [16] and a negative membrane potential [17]. Recently, we described that CyaA forms a calcium-conductive path in cell membrane and mediates influx of extracellular Ca^2+^ ions into cell cytosol concomitantly with translocation of the AC domain polypeptide into cells [18].

The current working model predicts that both Ca^2+^ influx and AC translocation depend on a different membrane-inserted CyaA conformer than the pore-forming activity [19], [20]. The two membrane activities of CyaA, however, appear to use the same essential amphipatic transmembrane segments within the pore-forming domain (α-helix_502–522_ and α-helix_565–591_), employing them in an alternative and mutually exclusive way. These segments harbor two pairs of negatively charged glutamate residues (Glu_509_/Glu_516_ and Glu_570_/Glu_581_) that were found to play a central role in toxin action on cell membrane. These control, respectively, the translocation of the positively charged AC domain, the formation of oligomeric CyaA pores and the cation-selectivity of the CyaA pore. Charge-reversing, neutral or helix-breaking substitutions of these glutamates were found to shift the balance between AC translocating and pore-forming activities of CyaA on cell membrane [8], [10], [19], [20].

The very high specific AC enzyme activity of CyaA allowed previously to detect its capacity to promiscuously bind and penetrate at reduced levels also numerous cell types lacking the CD11b/CD18 receptor [21], [22]. This is likely due to a weak lectin activity of CyaA, which would enable interaction of the toxin with cell surface gangliosides [23] and glycoproteins [24]. Indeed, binding of CyaA to CD11b/CD18 was recently found to depend on initial interaction with the N-linked glycan antenna of the receptor [24], where the specificity of CyaA for CD11b/CD18 appears to be determined by a segment of the stalk domain of the CD11b subunit (Osicka et al., manuscript in preparation).

CD11b/CD18 belongs to the β_2_ subfamily of polyfunctional integrins playing a major role in leukocyte function. The same β_2_ subunit (CD18) can, indeed, pair with four distinct α subunits to yield the α_L_β_2_ (CD11a/CD18, LFA-1), α_M_β_2_ (CD11b/CD18, CR3, Mac1), α_X_β_2_ (CD11c/CD18, p150/195) and α_D_β_2_ (CD11d/CD18) receptors, respectively [25]. Among key features of these integrins is their capacity of bi-directional signaling, where the avidity and conformation of the integrins is regulated by intracellular signals in the ‘inside-out’ signaling mode. In turn, binding of ligands or counter-receptors results in ‘outside-in’ signaling [26]. Among other effects, the latter yields actin cytoskeletal rearrangements and can result in lateral segregation of the β_2_ integrins from the bulk phase of the plasma membrane into distinct lipid assemblies known as lipid rafts [27], [28]. These were first detected as detergent-resistant membrane (DRM), characterized by insolubility in some detergents under certain conditions and enriched in cholesterol, sphingolipids, and glycosylphosphatidylinositol-anchored proteins [29]–[32]. Besides playing an important role in signal transduction, receptor internalization, vesicular sorting or cholesterol transport [33], the components of lipid rafts are often exploited as specific receptors mediating cell entry of toxins, pathogenic bacteria, or viruses [34]–[36].

Here, we show that CyaA-mediated influx of Ca^2+^ ions into cells induces mobilization of the toxin-receptor complex into lipid rafts, where translocation of the AC domain across cytoplasmic membrane is accomplished.

## Results

### CyaA entrains its CD11b/CD18 receptor into lipid rafts

**Figure 1:**
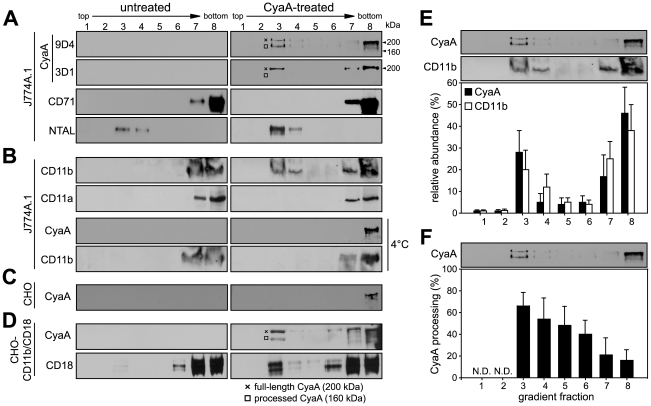
CyaA entrains CD11b/CD18 into lipid rafts. J774A.1 cells were mock-treated with buffer, or incubated with 1 nM CyaA at 37°C for 10 min (CyaA-treated). Cells were placed on ice and extracted at 4°C for 60 minutes in buffer containing 1% Triton X-100. Cell lyzates were fractionated by flotation in buoyant sucrose density gradients at 150,000×*g* in a Beckman SW60Ti rotor at 4°C for 16 h. Gradient fractions were analyzed by Western blotting. (A) Unless otherwise stated, CyaA was detected using the 9D4 monoclonal antibody (MAb) recognizing the C-terminal RTX repeats. The 3D1 MAb was used to specifically detect the distal part (aa 373–400) of the AC domain of CyaA [37]. The full-length CyaA (∼200 kDa) and the processed form of CyaA (∼160 kDa) are indicated by a crossline (×) and a square symbol (□), respectively. (B) CD11b was detected with the OKM-1 antibody on native immunoblots of Blue-native PAGE gels, while conventional Western blots were used for detection of CyaA and CD11a by 9D4 and MEM-25 antibodies, respectively. (C) Chinese hamster ovary (CHO) cells were mock-treated or incubated with 113 nM CyaA at 37°C for 10 min. (D) CHO cells stably transfected with genes encoding the human CD11b/CD18 integrin subunits (CHO-CD11b/CD18) were incubated with 1 nM CyaA at 37°C for 10 min. CyaA and CD18 were detected with 9D4 and MEM-48, respectively. Lanes 1–8 correspond to gradient fractions. Representative immunoblots from at least 3 independent experiments are shown. (E) Distribution of CyaA (filled bars) and CD11b (open bars) across sucrose density gradient. The values for relative amounts of CyaA and CD11b, detected in a given fraction of the gradient and expressed as % of total detected protein, were derived from densitometric analysis of immunoblots. (F) Relative abundance of the processed form of CyaA (160 kDa) was expressed as % of total CyaA detected within a given fraction of the gradient. Values represent the mean ± S.D. of three independent experiments. N.D., not determined.

To examine whether CyaA localizes to lipid rafts, murine J774A.1 monocytes exposed to 1 nM CyaA (176 ng/ml, 37°C, 10 min) were lyzed with ice-cold Triton X-100 and detergent-resistant membrane (DRM) was separated from soluble cell extracts by flotation through sucrose density gradients. As shown in Fig. 1A, while the CD71 marker of bulk membrane phase was exclusively detected in the soluble extract at the bottom of the gradient, up to 30% of total loaded CyaA was found to float in fraction 3 at a lower buoyant density towards the top of the gradient, together with the DRM marker protein NTAL (see Fig. 1E for quantification).

Notably, while over 50% of the full-length CyaA molecules (∼200 kDa) remained in the soluble phase at the bottom of the gradient, the floating DRM fractions were selectively enriched in a processed CyaA form of ∼160 kDa, representing up to 60% of total CyaA in the fraction 3 of the gradient (see Fig. 1F for quantification). This appeared to have the entire AC domain-cleaved off, as it could only be detected by the 9D4 antibody recognizing the C-terminal RTX repeats and not by the 3D1 antibody binding between residues 373 and 399 of the C-terminal end of the AC domain of CyaA [37]. Most CyaA molecules accumulating in DRM appeared, hence, to have the AC domain translocated across cellular membrane and accessible to processing by intracellular proteases [10], [38].

As further documented in Fig. 1B, no CD11b was floating with DRM from mock-treated J774A.1 cells, or when toxin binding occurred at 4°C. In turn, exposure of monocytes to 1 nM CyaA at 37°C, resulted in mobilization of over 30% of total cellular CD11b into the floating DRM, showing that CyaA relocated from the bulk of the membrane to DRM together with its α_M_β_2_ integrin receptor. This was, however, not due to any generalized clustering and mobilization of β_2_ integrins into rafts resulting from toxin action, as the highly homologous CD11a subunit of the other β_2_ integrin expressed by J774A.1 cells (LFA-1), was not mobilized into DRM (Fig. 1B). Hence, the relocation of CD11b/CD18 into rafts was specifically due to interaction with CyaA.

To assess whether CyaA association with DRM depended on toxin interaction with CD11b/CD18, we used Chinese hamster ovary (CHO) cells that do not express any β_2_ integrins unless transfected by genes encoding CD11b and CD18 subunits (CHO-CD11b/CD18). As shown in Fig. 1C, even when the CyaA concentration was raised to 113 nM, to obtain detectable amounts of the toxin associated with mock-transfected CHO cells, CyaA was detected exclusively in the soluble extract at the bottom of the gradient. In contrast, association of CyaA and CD11b/CD18 with DRM was detected already upon treatment of CHO-CD11b/CD18 transfectants with 1 nM CyaA (Fig. 1D). This showed that CyaA depended on binding to CD11b/CD18 for association with DRM and it was able to mobilize CD11b/CD18 into DRM independently of the myeloid cell background.

### Mobilization of the CyaA-CD11b/CD18 complex into lipid rafts depends on AC domain translocation and influx of extracellular calcium ions

**Figure 2:**
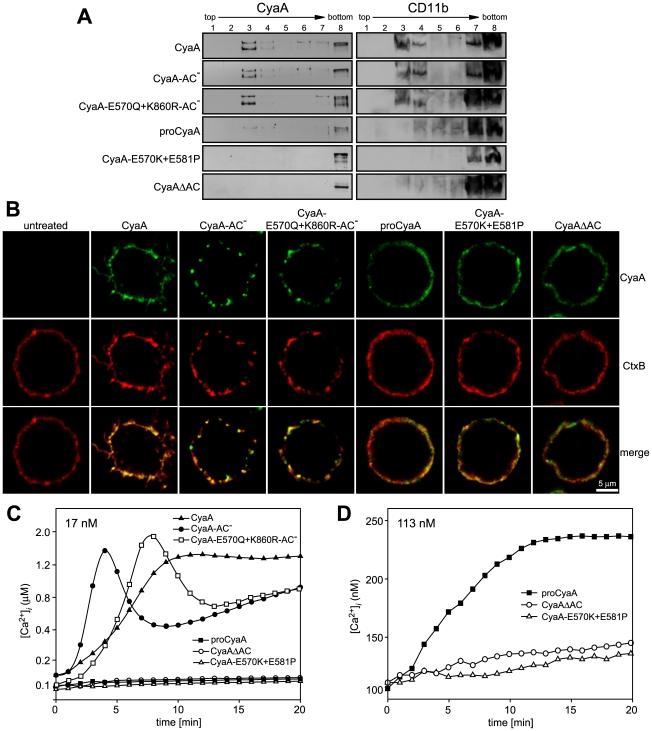
Mobilization of CyaA into lipid rafts depends on CyaA-mediated Ca^2+^ influx. (A) J774A.1 cells were incubated with 1 nM CyaA-derived proteins at 37°C for 10 min, cell lyzates were fractionated on sucrose gradients and proteins were detected by immunoblots as described for Fig. 1. (B) J774A.1 cells were incubated at 37°C for 10 min with 6 nM CyaA proteins labeled with Alexa Fluor 488 before 5 µg/ml of Alexa Fluor 594-labeled recombinant cholera toxin subunit B (CtxB) was added for additional 5 min. The cell-bound proteins were visualized by fluorescence microscopy and co-localization of CyaA (green) with CtxB (red) was assessed in the merged images (yellow). Representative images from two independent experiments are shown. Note that intact CyaA induced the reported cAMP-dependent ruffling of J774A.1 cells [2]. (C and D) CyaA induces increase of cytosolic calcium concentration ([Ca^2+^]*_i_*). J774A.1 cells were loaded with the Ca^2+^ probe Fura-2/AM (3 µM) at 25°C for 30 min and exposed to 17 nM (C) or 113 nM (D) CyaA proteins. Time course of Ca^2+^ entry was recorded as the ratio of fluorescence intensities (excitation at 340/380 nm, emmision 505 nm), as previously described [18]. The shown curves are representative of at least three independent experiments.

Since CyaA exerts several activities on cells in parallel, we analyzed which of them enabled mobilization of the CyaA-CD11b/CD18 complex into DRM. Towards this aim, we used a specific set of CyaA variants that retain the capacity to bind CD11b/CD18, while lacking one or more of the other CyaA activities (Table 1). As documented in Fig. 2A, the capacity of CyaA to elevate cellular cAMP concentrations was not required for mobilization of CyaA into DRM. The enzymatically-inactive CyaA-AC^−^ construct, unable to catalyze conversion of ATP into cAMP, was indeed accumulating in DRM with the same efficacy as the intact CyaA (fractions 3–4). Fatty-acylation of CyaA as such was also not essential for association of CyaA with DRM. As further shown in Fig. 2A, the non-acylated proCyaA was detected in DRM despite an importantly reduced capacity to associate with cells. Moreover, the pore-forming activity of CyaA was both insufficient and dispensable for mobilization of CyaA into DRM. The acylated CyaAΔAC construct, lacking the entire AC domain but retaining an intact pore-forming (hemolytic) capacity, was unable to mobilize into DRM (Fig. 2A). In contrast, the CyaA-E570Q+K860R-AC^−^ construct unable to permeabilize cells to any significant extent, but exhibiting an intact capacity to translocate the AC domain across cell membrane was, indeed, recruited into DRM together with CD11b/CD18 as efficiently as intact CyaA. In turn, the CyaA-E570K+E581P double mutant that was unable to form CyaA pores, or to translocate the AC domain across membrane, and retained only the CD11b-binding capacity (Table 1), was also unable to associate with DRM.

**Table 1:** Relative toxin activities of CyaA-derived constructs.

Protein	AC enzyme activity^a^	CD11b^+^ cell binding [%]^b^	Hemolytic activity [%]^c^	AC domain translocation [%]^d^	Calcium influx^e^
CyaA	+	100	100	100	+++
CyaA-AC^−^	−	N.D. (100^f^)	96±12	N.D. (100^g^)	+++
proCyaA	+	23±8	<1	3±1	+
CyaA-E570K+E581P	+	91±15	<1	1.8±0.5	−
CyaA-ΔAC	−	N.D. (100^f^)	109±15	−	−
CyaA-E570Q+K860R-AC^−^	−	N.D. (100^f^)	<1	N.D. (100^g^)	+++

a Capacity to catalyze conversion of ATP to cAMP.

b AC enzyme activity associated with J774A.1 cells (10^6^/ml) upon incubation with 6 nM protein for 30 min at 4°C. Relative activity of intact CyaA was taken as 100%.

c Determined as the amount of hemoglobin (A_541 nm_) released from washed sheep erythrocytes (5.10^8^/ml) by 5 µg/ml of protein at 37°C.

d Amounts of intracellular cAMP *per* 10^5^ J774A.1 cells incubated with indicated proteins for 30 min at 37°C.

e The number of plus signs reflects the relative ability of CyaA proteins to increase [Ca^2+^]_i_ levels (c.f. Fig. 2).

f Due to lack of AC enzyme activity, the capacity of these proteins to bind the J774A.1 cells could not be quantified directly. The capacity of these constructs to compete for the CD11b/C18 receptor with CyaA-biotin, however, is indistinguishable from that of intact CyaA (100% activity, data not shown).

g The capacity to translocate the AC domain polypeptide could not be quantified for these constructs because of lack of AC enzyme activity. It can, however, be deduced from the capacity of corresponding constructs to deliver an inserted OVA epitope (SIINFEKL) into the cytosol of antigen-presenting cells for processing and subsequent presentation to specific CD8^+^ T cells in complex with major histocompatibility complex I molecules [63].

To corroborate these observations, we used fluorescence microscopy to examine the distribution of individual CyaA proteins in cell membrane. As documented in Fig. 2B, the intact CyaA, CyaA-AC^−^ and CyaA-E570Q+K860R-AC^−^ proteins were found to induce formation of, and to localize within, patches on cell membrane. Moreover, the same patches were labeled to high extent also with B subunit of cholera toxin (CtxB), which specifically binds the GM1 ganglioside accumulating in lipid rafts. Hence, the three CyaA variants capable of associating with DRM (cf. Fig. 2A) were also found to co-localize with CtxB within membrane patches. In turn, no formation of membrane patches, a diffuse distribution on cell surface, and low if any co-localization with CtxB, were observed for the CyaAΔAC and CyaA-E570K+E581P constructs that were unable to associate with DRM, too.

The pattern of DRM association, processing to the 160 kDa form and co-localization of the different CyaA variants with CtxB, respectively, resembled strongly the pattern of structure-function relationships observed recently for the capacity of CyaA to promote influx of extracellular calcium ions into J774A.1 cells [18]. Indeed, as documented in Fig. 2C by measurements of intracellular calcium concentrations ([Ca^2+^]*_i_*), the CyaA, CyaA-AC^−^ and CyaA-E570Q+K860R-AC^−^proteins (17 nM) exhibited an expected capacity to promote Ca^2+^ influx into J774A.1 cells (see [18] for details on different kinetics of Ca^2+^ entry for AC^−^ and AC^+^ constructs). In contrast, the CyaA-E570K+E581P and CyaAΔAC constructs, failed to mediate any increase of [Ca^2+^]*_i_* even when used at a 113 nM concentration (Fig. 2D). Collectively, hence, these results show that the capacity of different CyaA variants to associate with DRM and co-localize with CtxB within coalesced lipid rafts was mirrored by the capacity to promote Ca^2+^ influx into cells.

### Membrane insertion of a ‘translocation intermediate’ allows calcium influx and CyaA association with DRM

**Figure 3:**
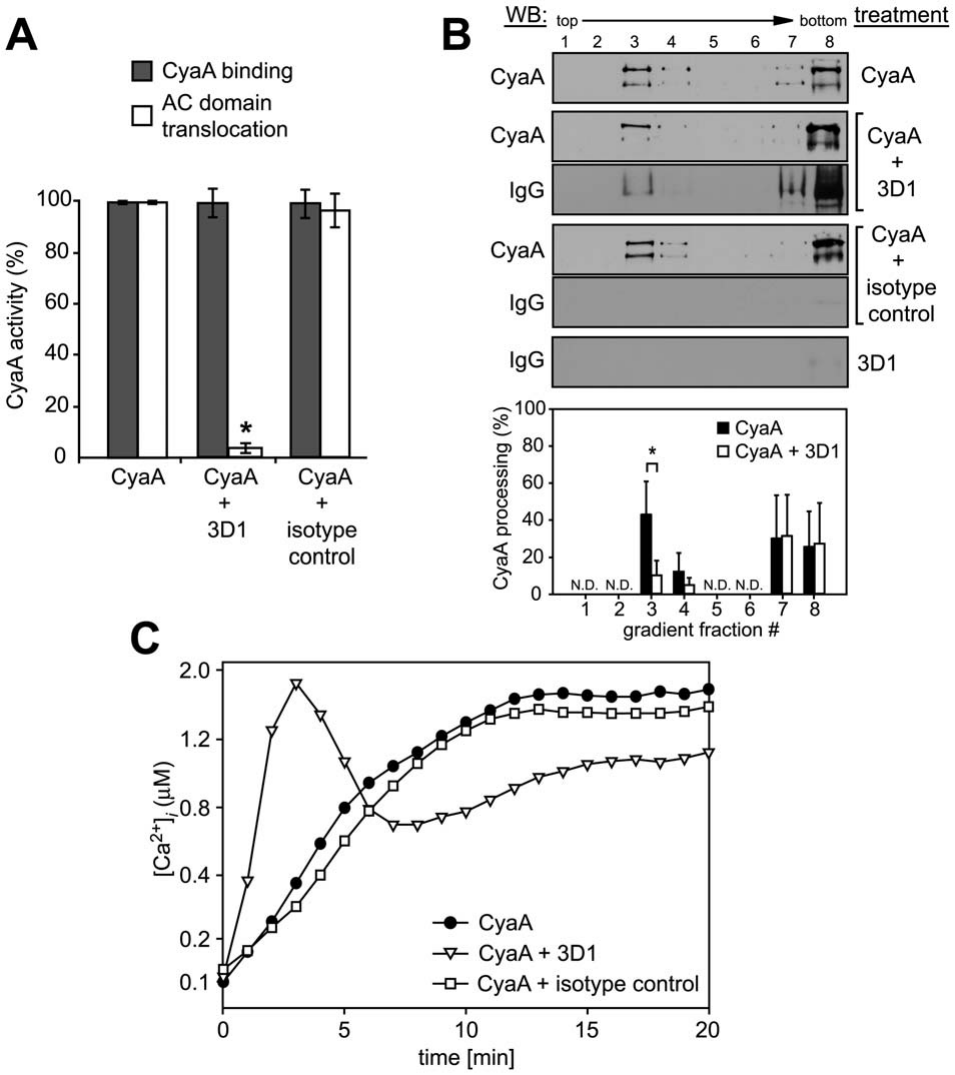
Binding of 3D1 antibody uncouples AC translocation from membrane insertion and CyaA-mediated Ca^2+^ influx. (A) 17 nM CyaA was preincubated for 20 min at 37°C with 20 µg/ml of 3D1 MAb (CyaA+3D1) or the Tu-01 IgG1 isotype control MAb (CyaA+isotype control), before 6 nM toxin was added to J774A.1 cells. CyaA binding was determined as the amount of total cell-associated AC enzyme activity upon cells incubation with 6 nM CyaA for 10 min at 37°C in the presence or absence of the indicated antibody. AC domain translocation was assessed by determining the intracellular concentration of cAMP generated in cells in the presence or absence of the indicated antibodies, following incubation of cells with four different toxin concentrations from within the linear range of the dose-response curve (0.05, 0.1, 0.25, and 0.5 nM). The % of cAMP accumulation in cells at each toxin concentration was calculated, taking cAMP values for CyaA preincubated in buffer alone as 100%. The average of such determined % activity values is given. An *asterisk* indicates a statistically significant difference (*, *p*<0.01; Student's *t* test). (B) J774A.1 cells were exposed to 1 nM CyaA alone, or to 1 nM CyaA preincubated with 3D1 or IgG1 isotype MAb as above. Cell lyzates were separated on sucrose density gradients and analyzed as in Fig. 1. The 3D1 and isotype IgG1 MAbs were detected with anti-mouse IgG antibody. The relative amounts of processed CyaA (∼160 kDa) within individual gradient fractions were determined as in Fig. 1F. Values represent the mean ± S.D. of three independent experiments. An *asterisk* indicates a statistically significant difference (*, *p*<0.05; Student's *t* test). N.D., not determined. (C) J774A.1 cells were loaded with Fura-2/AM as above and exposed to 17 nM CyaA alone, or to CyaA preincubated with 3D1 or an IgG1 isotype control MAb. Ca^2+^ influx was recorded as above and the shown curves are representative of three independent experiments.

We showed recently that CyaA-mediated influx of Ca^2+^ into cells is independent of the AC enzyme or pore-forming (hemolytic) activities of CyaA and occurs concomitantly to translocation of the AC domain across target cell membrane [18]. It remained, however, to assess whether it was the mere insertion of a CyaA translocation precursor into cell membrane, or whether the accomplishment of translocation of the AC domain across cell membrane was required for formation of a calcium conductive path in cell membrane. Towards this aim, we used the 3D1 monoclonal antibody (MAb) that binds to the distal end of the AC domain (residues 373 to 400) and was previously shown to block membrane translocation of the AC domain of cell-associated CyaA [39]. As expected and documented in Fig. 3A, preincubation of CyaA with the 3D1 MAb did not affect the capacity of CyaA to bind J774A.1 cells, while strongly inhibiting AC domain delivery and cAMP concentration elevation in cells. However, as revealed by detection of both CyaA and 3D1 in the fraction 3 of the sucrose gradient shown in Fig. 3B, the membrane-inserted CyaA with bound 3D1 MAb still associated with DRM at the same levels as CyaA alone. Moreover, as also shown in Fig. 3B, due to 3D1-mediated inhibition of AC domain translocation, the relative amount of processed CyaA (∼160 kDa) in the DRM fractions decreased to 10 to 15% of total present CyaA, while over 50% of CyaA in DRM was processed in the presence of isotype control.

As shown in Fig. 3C, however, despite arrested AC domain translocation, the CyaA-3D1 complex promoted elevation of [Ca^2+^]*_i_* in cells with kinetics resembling the Ca^2+^ influx produced by CyaA-AC^−^ (cf. Fig. 2C). Hence, 3D1 binding uncoupled translocation of the AC domain from membrane insertion of CyaA and ‘locked’ the toxin in the conformation of a ‘translocation intermediate’ that permeabilized cells for Ca^2+^ ions and associated with DRM.

### Influx of Ca^2+^ induces association of CyaA with DRM

**Figure 4:**
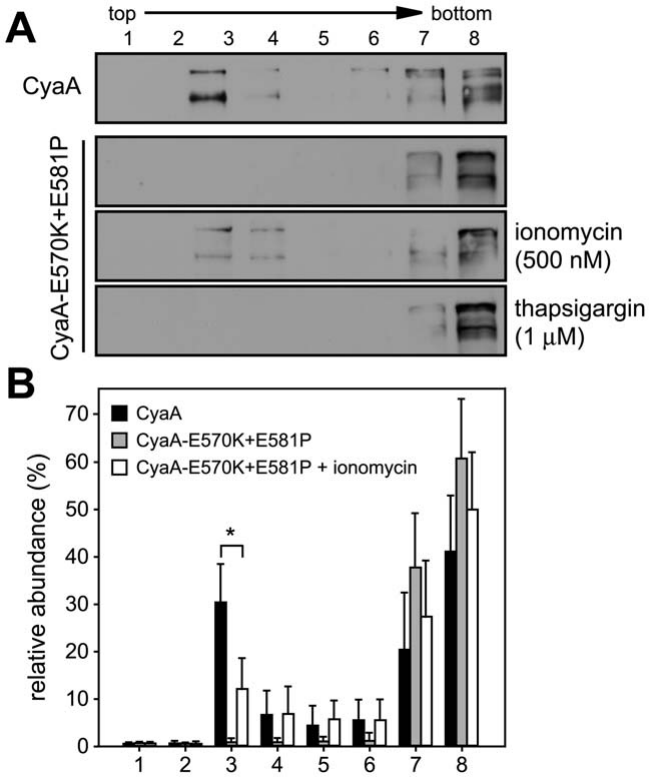
Mobilization of CyaA into lipid rafts depends on influx of extracellular Ca^2+^ ions into cells. (A) J774A.1 cells were incubated at 37°C for 10 min with 1 nM CyaA and CyaA-E570K+E581P, in the presence or absence of 500 nM ionomycin , or of 1 µM thapsigargin, respectively. Cell lyzates were analyzed on sucrose density gradients as above. The blots are representative of four independent experiments. (B) Distribution of CyaA proteins in gradient fractions was quantified as in Fig. 1 and the values represent the mean ± S.D. of three independent experiments. An *asterisk* indicates a statistically significant difference (*, *p*<0.05; Student's *t* test).

Next, we aimed to determine whether elevation of [Ca^2+^]*_i_* as such would mobilize into DRM also the CyaA-E570K+E581P protein unable to associate with DRM on its own (cf. Fig. 2). As demonstrated in Fig. 4, upon permeabilization of cells for extracellular Ca^2+^ ions with the Ca^2+^ ionophore ionomycin (500 nM), up to 15% of the added CyaA-E570K+E581P was found associated with DRM. In contrast, no association of CyaA-E570K+E581P with DRM was observed upon treatment of cells with 1 µM thapsigargin that increases [Ca^2+^]*_i_* by triggering Ca^2+^ release from intracellular stores. This showed that entry of extracellular Ca^2+^ across the cytoplasmic membrane was required for mobilization of CyaA into DRM.

### Recruitment of CyaA-CD11b/CD18 complexes into rafts depends on cleavage of cytosolic talin by the Ca^2+^-activated protease calpain

**Figure 5:**
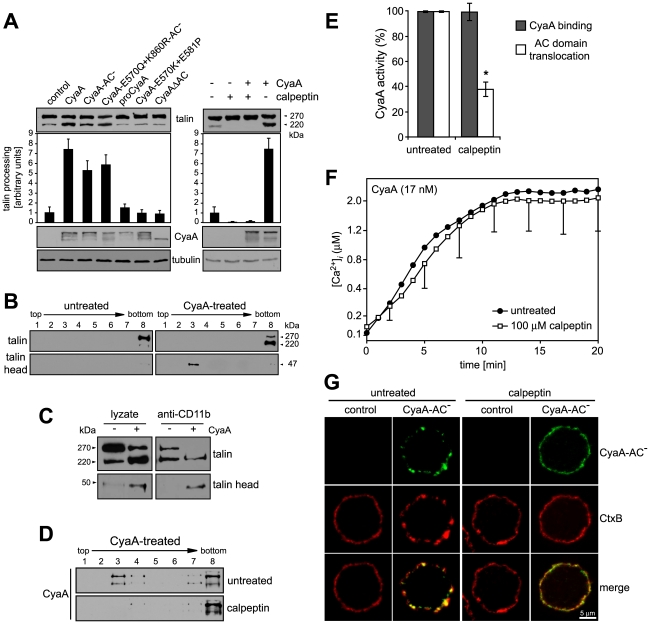
Mobilization of CyaA into lipid rafts depends on talin cleavage by calpain. (A) J774A.1 cells were incubated with 30 nM CyaA proteins at 37°C for 10 min (left panel). Talin, CyaA and α-tubulin were detected in cell lyzates by Western blotting using 8d4, 9D4 and Tu-01 MAbs, respectively. Calpain activation was inhibited by preincubation of cells with 100 µM calpeptin at 37°C for 30 min (right panel). Talin processing was determined from Western blots as relative increase of the amount of the 220-kDa talin fragment, taking the value for untreated cells as 1. Data represent the mean ± S.D. from three experiments. (B) Cells were kept in buffer or pretreated with 100 µM calpeptin at 37°C for 30 min before incubation with 1 nM CyaA at 37°C for 10 min. Talin, talin head and CyaA were detected in cell lyzates separated on sucrose density gradients using 8d4, TA-205 and 9D4 MAbs, respectively. The shown blots are representative of four independent experiments. (C) CyaA-mediated talin cleavage promotes CD11b/CD18 release from cytoskeletal constraints. J774A.1 cells were incubated with 30 nM CyaA for 30 min at 37°C. Cell lyzates were prepared and incubated with anti-CD11b monoclonal antibody (MEM-174) covalently coupled to CNBr-activated Sepharose beads. The beads were washed, bound proteins were eluted with SDS-PAGE loading buffer, and talin and talin head were detected by Western blotting with 8d4 and TA-205 antibody, respectively. (D) DRM association of CyaA in J774A.1 cells kept in buffer or preincubated with 100 µM calpeptin was analyzed as above. (E) AC translocation into control and calpeptin-pretreated J774A.1 cells were determined as described in the legend to Fig. 3. (F) CyaA-mediated influx of Ca^2+^ into untreated and calpeptin-pretreated J774A.1 cells was measured as in Fig. 2. Values represent the mean ± S.D. of three independent experiments. An *asterisk* indicates a statistically significant difference (*, *p*<0.05; Student's *t* test). (G) J774A.1 cells grown on coverslips were kept in buffer or pretreated with 100 µM calpeptin at 37°C for 30 min before membrane distribution of fluorescently labeled CyaA-AC^−^ (6 nM, green) and CtxB (5 µg/ml, red) was visualized as described in the legend to Fig. 2.

Influx of extracellular Ca^2+^ during leukocyte activation was reported to induce mobilization of integrins in cell membrane by calpain-mediated cleavage of talin that tethers β2 integrins to actin cytoskeleton [40], [41]. Therefore, we examined whether CyaA-promoted recruitment of the toxin receptor into rafts depended on talin processing. As shown in Fig. 5A, intact talin (∼270 kDa) was largely predominating in lyzates of cells treated with the CyaA-ΔAC or CyaA-E570K-E581P proteins that are unable to promote Ca^2+^ entry into cells. In contrast exposure of cells to the CyaA, CyaA-AC^−^, or CyaA-E570Q+K860R-AC^−^ proteins, promoting influx of Ca^2+^ into cells, increased about seven-fold the detected amounts of the ∼220 kDa C-terminal fragment of processed talin (Fig. 5A, left panel). Concomitantly, increased amounts of the 47-kDa N-terminal fragment of talin (talin head) were detected in cell lyzates. Moreover, tightly associated talin head was found to float together with the CD11b/CD18 heterodimer in DRM (Fig. 5B) and could be co-immunoprecipitated with the integrin on beads coated with anti-CD11b antibody (Fig. 5C). This CyaA-induced processing of talin was clearly due to activation of calpain, as preincubation of cells with 100 µM calpain inhibitor, calpeptin, blocked talin cleavage in CyaA-treated cells (Fig. 5A, right panel). Remarkably, pretreatment of cells with calpeptin strongly inhibited also the association of CyaA with DRM (Fig. 5D) and decreased by at least a factor of two the capacity of cell-associated CyaA to translocate the AC enzyme into target cells (Fig. 5E). In turn, no effect of calpain inhibition was observed for CyaA-mediated Ca^2+^ influx (Fig. 5F). In line with these results, pretreatment of cells with 100 µM calpeptin blocked effectively also the formation of CyaA-AC^−^ patches in cell membrane and ablated co-localization of CyaA-AC^−^ with CtxB, as documented in Fig. 5G. It can, hence, be concluded that CyaA-mediated influx of Ca^2+^ into cells activated cleavage of talin by calpain and this was required for mobilization of CyaA-CD11b/CD18 complexes into lipid rafts.

### Ca^2+^ influx, lipid raft association and AC translocating activity of CyaA depend on cholesterol content of target cell membrane

**Figure 6:**
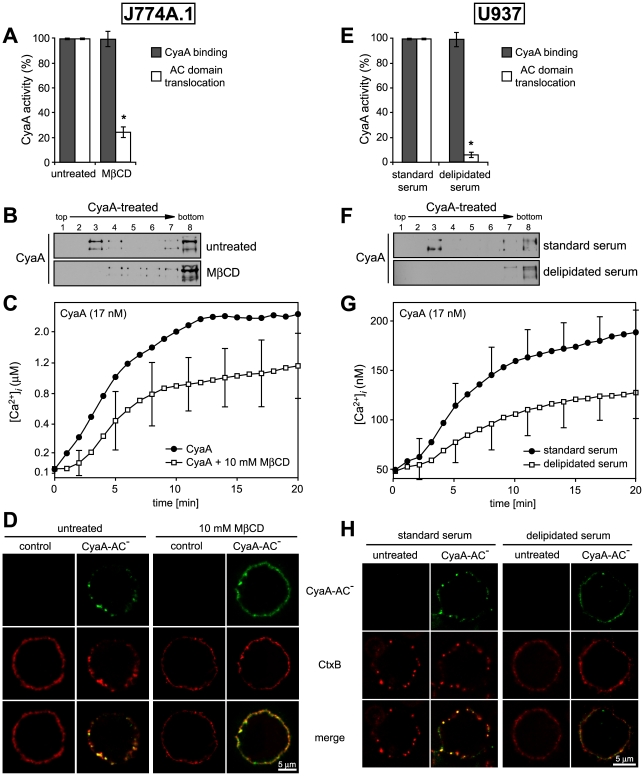
Cholesterol depletion inhibits translocation of AC domain across target cytoplasmic membrane. (A) J774A.1 cells were kept in buffer or treated with 10 mM MβCD at 37°C for 30 min, before CyaA was added for additional 10 min. CyaA binding and AC domain translocation were determined as described in the legend to Fig. 3. Values represent the mean ± S.D. from four independent experiments performed in triplicates. An *asterisk* indicates a statistically significant difference (*, *p*<0.01; Student's *t* test). (B) J774A.1 cells were kept in buffer or pretreated with 10 mM MβCD at 37°C for 30 min and incubated with 1 nM CyaA for 10 min. Cell lyzates were analyzed as in Fig. 1A. (C) J774A.1 cells were loaded with the Ca^2+^ probe Fura-2/AM prior to cholesterol extraction with 10 mM MβCD at 30°C for 30 min. CyaA-mediated Ca^2+^ influx was recorded as above. Standard deviations were calculated for mean values at indicated time points and the shown curves are representative of at least three independent experiments. (D) J774A.1 cells grown on coverslips were kept in buffer or pretreated with 10 mM MβCD at 37°C for 30 min. Membrane distribution of fluorescently labeled CyaA-AC^−^ (6 nM, green) and CtxB (5 µg/ml, red) was visualized as described in the legend to Fig. 2. (E) U937 cells were grown for 48 hours in RPMI medium supplemented with 10% fetal calf serum (standard serum) or with 10% lipoprotein-deficient serum from fetal calf (delipidated serum). CyaA binding and AC domain translocation were determined as described in the legend to Fig. 3. Values represent the mean ± S.D. from four independent experiments performed in triplicates. An *asterisk* indicates a statistically significant difference (*, *p*<0.01; Student's *t* test). (F) U937 cells grown in standard or delipidated serum were incubated with 1 nM CyaA at 37°C for 10 min, cell lyzates were separated on sucrose density gradients and analyzed as in Fig. 1. (G) U937 cells grown in standard or delipidated serum were loaded with the Ca^2+^ probe Fura-2/AM (3 µM) at 25°C for 30 min, exposed to CyaA (17 nM) and the time course of Ca^2+^ entry was recorded as above. Standard deviations were calculated for mean values at indicated time points and the shown curves are representative of at least three independent experiments. (H) U937 cells cultured with standard or delipidated serum were mounted on polylysin-coated coverslips and membrane distribution of fluorescently labeled CyaA-AC^−^ (6 nM, green) and CtxB (5 µg/ml, red) was visualized as described above.

To determine what role does association of CyaA with lipid rafts play in the mechanism of toxin action on cellular membrane, we analyzed the activities of CyaA on cells having the rafts disrupted by depletion of cholesterol. As shown in Table 2, the total cholesterol content of J774A.1 cells could be decreased about two-fold by cholesterol extraction with 10 mM MβCD for 30 min. While the disruption of raft structures did not impact on association of CyaA with cells (Fig. 6A and Fig. S1), the modest decrease of cellular cholesterol content yielded an about five-fold decrease of the capacity of CyaA to translocate the AC domain across cell membrane. This defect was further mirrored by decreased DRM association of CyaA in MβCD-extracted cells, as shown in Fig. 6B. In parallel, the specific capacity of CyaA to promote Ca^2+^ influx into cholesterol-depleted cells was reduced and the [Ca^2+^]*_i_* increase ensuing toxin addition was delayed by several minutes, reaching a plateau at about a half-maximal [Ca^2+^]*_i_* concentration, as compared to non-depleted cells (Fig. 6C). In line with this, the two-fold decrease of cellular cholesterol level moderately decreased also the co-localization of CyaA with CtxB in lipid rafts (Fig. 6D).

**Table 2:** Cholesterol content in J774 and U937 cells.

cells	ng of cholesterol *per* 10^6^ cells	
	untreated	extracted
J774A.1	290±35	140±43
U937 - normal serum	89±12	47±8
U937 - delipidated serum	8±3	N.D.

Cholesterol was extracted from cells using 10 mM MβCD at 37°C for 30 min. U937 cells deficient in endogenous cholesterol synthesis were grown in RPMI medium supplemented with 10% normal fetal calf serum or with 10% lipoprotein-deficient serum from fetal calf (delipidated serum) for 48 h. Cholesterol content was determined using the Amplex Red Cholesterol Assay Kit (Molecular Probes). Values represent the mean ± S.D. obtained from three independent experiments. N.D., not detectable.

Therefore, the above described experiments were replicated on monocytic U937 histiocytic lymphoma cells (CD11b^+^) that are defective in endogenous cholesterol synthesis. These cells can be efficiently depleted of cholesterol without losing viability, by them growing for 48 hours in media containing cholesterol-free (delipidated) serum. As shown in Table 2, such treatment reduced the cholesterol content of U937 cells almost 10-times.

As shown in Fig. 6E, a pronounced, over ten-fold decrease of specific AC translocation capacity of CyaA was observed on U937 cells grown in media with delipidated serum, as compared to CyaA activity on cells grown with standard serum. At the same time, however, the total amounts of cell-associated CyaA remained equal, irrespective of cell treatment. However, by difference to well-detectable DRM association of CyaA on cholesterol-replete U937 cells, grown with standard serum, no association of CyaA with DRM was observed in lyzates of cholesterol-depleted U937 cells grown in delipidated serum, respectively (Fig. 6F).

Intriguingly, compared to the Ca^2+^ influx elicited by equal concentrations of CyaA in J774A.1 cells, about an order of magnitude lower amplitude and delayed kinetics of CyaA-mediated Ca^2+^ influx was observed for U937 cells grown in media with standard serum (cf. Fig. 6C and Fig. 6G). These cells exhibited a 3-fold lower cholesterol content than the J774A.1 cells (see Table 2), suggesting that the low cholesterol content of U937 cells might have accounted for the poor capacity of CyaA to elicit Ca^2+^ influx in these cells. Indeed, when cholesterol content of J774A.1 cells was reduced about two-fold by cholesterol extraction with 10 mM MβCD, a delayed kinetics of CyaA-induced influx of Ca^2+^ into J774A.1 cells and a two-fold lower final [Ca^2+^]*_i_* reached in 20 minutes, were also observed (cf. Fig. 6C). Similarly, a delayed influx of Ca^2+^ and a lower final level of [Ca^2+^]*_i_* was observed also upon addition of equal CyaA concentrations to U937 cells depleted of cholesterol by growth in delipidated media, as compared to U937 cells grown in standard media, as shown in Fig. 6G. At the same time, however, the respective amounts of CyaA associated per 10^6^ J774A.1 or U937 cells remained the same (∼5 ng of CyaA bound per 10^6^ cells), irrespective of whether the cholesterol content of cells was decreased by the treatments (cf. Fig. 6A and 6E). These results, hence, strongly point towards a close relation between the overall content of cholesterol in cellular membrane and the propensity of the membrane-inserted CyaA to adopt the ‘translocation intermediate’ conformation, which would account for the Ca^2+^ conducting path across cell membrane (cf. Fig. 3 and [18]).

Finally, a correspondingly reduced CtxB binding and little if any co-localization of CtxB with CyaA were observed on cholesterol-depleted U937 cells, grown in delipidated serum, as compared to binding and some observable co-localization of CyaA with CtxB on cholesterol-replete U937 cells (Fig. 6).

### Mobilization of CyaA into lipid rafts enables translocation of AC domain across membrane

In the light of the above results, we aimed to test the hypothesis that AC translocation across membrane was supported and accomplished upon recruitment of the membrane-associated toxin into the cholesterol-rich environment of lipid rafts. Therefore, we examined whether the inactive CyaA-E570K+E581P construct would gain any capacity to translocate its enzymatically active AC domain across cellular membrane upon mobilization into lipid rafts. Since this mutant is intact for receptor binding but fails to promote Ca^2+^ influx into cells, we reasoned that mobilizing Ca^2+^ ions into cells *in trans*, by co-incubation with a translocating CyaA-AC^−^ toxoid, might promote recruitment of CyaA-E570K+E581P mutant into rafts to some extent.

**Figure 7:**
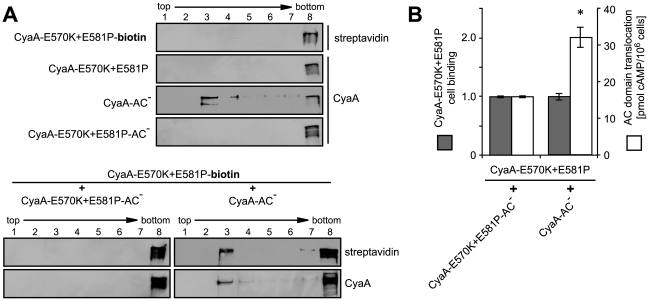
AC domain of CyaA translocates across cellular membrane from lipid rafts. (A) J774A.1 cells were incubated at 37°C for 10 min with 1 nM individual CyaA proteins (upper panel) or with their 1∶1 mixtures (lower panel). CyaA and biotinylated CyaA-E570K+E581P (CyaA-E570K+E581P-biotin) were detected in gradient fractions using 9D4 and streptavidine, respectively. (B) J774A.1 cells were incubated at 37°C for 10 min with the indicated pairs of proteins (12 nM) mixed in a 1∶1 molar ratio. Binding of CyaA-E570K+E581P-biotin was determined as the amount of total cell-associated AC enzyme activity upon exposure of cells to the protein mixtures and expressed as relative value. Enhancement of the residual capacity of CyaA-E570K+E581P to translocate AC domain into J774A.1 cell cytosol (1.8±0.5% of intact CyaA activity) was measured by determination of intracellular cAMP amounts accumulated in 10^6^ cells upon exposure for 10 min at 37°C to the indicated 1∶1 protein mixtures (12 nM). The values represent the mean ± S.D. from four independent experiments performed in duplicate. An *asterisk* indicates a statistically significant difference (*, *p*<0.01; Student's *t* test).

As shown in Fig. 7A, when biotinylated CyaA-E570K+E581P was added to cells alone, or when it was co-incubated with equal amounts of the enzymatically inactive CyaA-E570K+E581P-AC^−^ toxoid, unable to cause calcium influx, the CyaA-E570K+E581P-biotin failed to associate with DRM. In contrast, upon co-incubation with equal amounts of the translocating CyaA-AC^−^ toxoid (1∶1), a significant fraction of CyaA-E570K+E581P-biotin associated with DRM. Moreover, as shown in Fig. 7B, this mobilization into DRM was paralleled by a doubling of the residual capacity of the CyaA-E570K+E581P variant to deliver the AC domain across cell membrane and elevate cytosolic cAMP concentrations (Fig. 7B). Thus, recruitment into cholesterol-rich lipid rafts enhanced the residual AC translocating activity of this defective CyaA variant.

## Discussion

**Figure 8:**
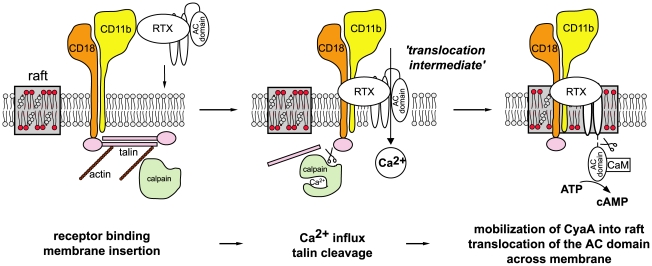
Model of CyaA translocation across target cell membrane. In the first step, CyaA binds the CD11b/CD18 integrin receptor dispersed in the bulk of the membrane phase outside of lipid rafts, having the cytoplasmic tail of the CD18 subunit tethered to actin cytoskeleton *via* the linker protein talin. Upon receptor engagement, a ‘translocation intermediate’ of CyaA inserts into the lipid bilayer of cell membrane with the AC domain partially penetrating into cell membrane together with the pore-forming segments of the toxin and participating in formation of a transiently opened Ca^2+^-conducting path across cell membrane. Influx of external calcium ions into cells induces activation of the Ca^2+^-dependent protease calpain, yielding talin cleavage and liberation of the CyaA-CD11b/CD18 complex from binding to actin cytoskeleton. Consequently, CyaA is recruited with CD11b/CD18 into cholesterol-enriched lipid rafts, where the liquid-ordered packing of lipids and the specific presence of cholesterol enable completion of AC domain translocation across cytoplasmic membrane. Upon exposure at the cytosolic side of cell membrane, the AC domain is cleaved-off from the RTX cytolysin moiety of CyaA by a protease residing inside the cell. Binding of cytosolic calmodulin (CaM) then activates the AC enzyme and unregulated conversion of ATP to cAMP is catalyzed.

We show here that membrane translocation of the adenylate cyclase domain of CyaA occurs by a two step mechanism and involves toxin piggybacking on the α_M_β_2_ integrin for relocation into lipid rafts. The present results allow us to propose a new model of CyaA mechanism of action, as summarized in Fig. 8. Upon initial binding of CyaA to the CD11b/CD18 receptor distributed in the bulk phase of cell membrane, a ‘translocation intermediate’ of CyaA would insert into the cytoplasmic membrane. It is assumed that in this ‘translocation intermediate’ a part of the AC domain is already inserted within the membrane and is shielded form the lipids by association with the amphipathic α-helical transmembrane segments of the hydrophobic domain of CyaA (residues 502–522, 529–549, 571–591, 607–627 and 678–698 [19], [20]). This ‘translocation intermediate’ then forms a path conducting external Ca^2+^ ions across cellular membrane into the submembrane compartment of cells. Incoming calcium ions activate the Ca^2+^-dependent protease calpain, located in the submembrane compartment, which produces cleavage of the talin tether. This liberates the toxin-receptor complex from association with actin cytoskeleton and mobilizes it for recruitment into lipid rafts. Within the specific liquid-ordered environment of cholesterol-rich lipid rafts, translocation of the positively charged AC domain across the cellular membrane is completed, driven by the negative gradient of membrane potential.

Deciphering this fine-tuned mechanism of toxin action on cell membrane fosters our understanding of the key role played by CyaA in virulence of *Bordetella*e during the early phases of bacterial colonization of host respiratory mucosa. It allows to propose the following scenario. The produced CyaA targets the CD11b/CD18 receptor of incoming myeloid phagocytic cells, such as neutrophils, macrophages and dendritic cells [42]. As CyaA action does not depend on receptor-mediated endocytosis, the toxin recruited into lipid rafts can rapidly translocate its highly active AC enzyme domain across the cytoplasmic membrane of cells, in a process exhibiting a half-time of only about ∼30 seconds [38]. Mobilization of toxin-receptor complexes into lipid rafts than promotes their clustering and potentially induces recruitment of cellular cAMP-responding elements, such as the protein kinase A anchored to AKAPs, the specific A-kinase anchoring scaffolds [43]–[45]. This would allow maximization of toxin action through subversive cAMP production in close vicinity of components of the cAMP-regulated PKA signaling pathway. This capacity to hijack the spatio-temporal regulation of cellular cAMP/PKA signaling would then endow CyaA with the high potency in paralyzing the central bactericidal mechanisms employed by myeloid phagocytic cells. Indeed, few picomoles of CyaA (1 ng/ml or less) were previously reported to instantaneously suppress the oxidative burst capacity of neutrophils [46], or the phagocytosis of complement-opsonized particles by macrophages [2].

Several other bacterial protein toxins appear, indeed, to utilize lipid rafts as a portal of cell entry, exploiting as specific receptors directly certain raft components, such as cholesterol, sphingolipids or GPI-anchored proteins [47]–[50]. In contrast, we found here that CyaA associates with rafts only upon binding and mobilization (hijacking) of its receptor CD11b/CD18. Unless activated in the process of leukocyte activation, this β_2_ integrin is distributed diffusely over the entire cellular membrane. As outlined above, we show here that upon binding of CyaA the integrin relocates into lipid rafts, due to toxin-induced and calcium-activated cleavage of talin by calpain. Moreover, the recently discovered capacity of CyaA to bind N-linked oligosaccharides of CD11b/CD18 [24] might also play a role in this process. It is, indeed, plausible to propose that CyaA interaction with terminal sialic acid residues of glycan chains of raft sphingolipids might also be contributing to accumulation of the CyaA-CD11b/CD18 complex in lipid rafts, as well as it may contribute to clustering of lipid rafts containing CyaA later-on. An evidence for CyaA interactions with gangliosides can, indeed, be deduced from the previously observed inhibition of CyaA activity on macrophages by the presence of micromolar concentrations of free gangliosides, such as G_T1b_ [23].

It remains to be addressed in future studies if CyaA can form oligomeric pores also once engaged in interaction with the target cell membrane through binding of the CD11b/CD18 receptor and whether CyaA can form pore-forming oligomers also in phagocyte membrane. We have recently succeeded in demonstrating the presence of the long-predicted CyaA oligomers within the membrane of cells lacking the receptor CD11b/CD18, such as erythrocytes [10]. Vojtova-Vodolanova with co-authors (2009), indeed, showed that formation of CyaA oligomers underlies the pore-forming activity of CyaA towards erythrocytes. However, despite significantly higher amounts of CyaA binding per single phagocyte cell through the CD11b/CD18 receptor, fairly high concentrations (>1 µg/ml) of the recombinant enzymatically inactive but fully pore-forming CyaA-AC^−^ variants are needed to provoke lysis of cells like J774A.1 monocytes in several hours [8]. While this resistance to colloid-osmotic lysis is likely to be to large extent due to membrane recycling mechanisms and pore removal form phagocyte cytoplasmic membrane, it remains to be shown that CyaA can form oligomeric pores in leukocyte membrane as well.

The results presented here do not indicate any role of CyaA oligomers in promoting calcium influx, toxin mobilization into rafts, or AC enzyme translocation into CD11b^+^ phagocytes. Early dose-dependence studies indicated that the AC domain was delivered across target cell membrane by CyaA monomers. Indeed, toxin molecules with the AC domain cleaved-off by cytosolic proteases, upon AC translocation into cells, were detected exclusively in form of CyaA monomers within erythrocyte membranes and were excluded from the detected CyaA oligomers [10]. Moreover, we used here the CyaA-E570Q+K860R-AC^−^ protein, which essentially lacks any pore-forming activity and fails to permeabilize the membrane of J774A.1 cells, thus being unlikely to form any CyaA oligomers (Table 1 and [51]). On the other hand, this construct is fully capable to translocate the AC domain into cytosol of CD11b-expressing J774A.1 cells, to promote calcium influx and to associate with DRM, or to co-localize with CtxB in coalesced rafts, respectively (cf. Fig. 2). It appears, therefore, unlikely that oligomerization plays a role in DRM association of CyaA.

We also observed here that the levels of binding of CyaA to CD11b-expressing cells were not affected upon cholesterol depletion of cell membrane, while the translocation of the AC domain across the membrane depended strongly on the cholesterol content. This suggests that by modulating the physical properties lipid bilayers, cholesterol was specifically supporting the translocation of the AC domain across cell membrane. Indeed, cholesterol removal was previously found to impair the residual penetration capacity of CyaA on artificial membranes and erythrocytes [52], [53]. This goes well with the impact of cholesterol concentration on membrane fluidity, lateral phase separation, formation of liquid-ordered structures and the propensity of lipids to adopt the inverted hexagonal phase [54], [55]. The same membrane properties would also be expected to support AC domain translocation into cells by lowering the energy barrier for polypeptide penetration into and across the lipid bilayer [56]. It is plausible to speculate that membrane translocation of the AC domain requires the presence of cholesterol-dependent liquid-ordered (l_o_) phase, in which the acyl chains of lipids are tightly packed, while the individual lipid molecules have a high degree of lateral mobility. The relative mobility of lipids in l_o_ domains represents, indeed, a likely prerequisite for passage of the AC domain across lipid bilayer. A high condensation and immobility of lipids in liquid-disordered (l_d_)-phase domains would, in turn, be expected to interfere with AC polypeptide translocation. The requirement for sufficient membrane fluidity for AC translocation to occur is also indicated by the block of AC translocation at 4°C [38].

Recently, we demonstrated that AC domain translocation across target cell membrane is accompanied by entry of Ca^2+^ ions into cells. Moreover, the AC domain polypeptide as such was found to participate in formation of the transiently opened calcium influx path in cell membrane [18]. Here, we used the 3D1 MAb recognizing a distal segment of the AC domain and show that blocking of AC domain translocation across cell membrane can lock CyaA in a ‘translocation intermediate’ conformation that forms a path for Ca^2+^ influx across cell membrane (cf. Fig. 3). Moreover, this ‘translocation intermediate’ was found to be recruited into lipid rafts (Fig. 3). The sum of the data hence allows us to answer the question what happens first, whether calcium influx precedes toxin mobilization into rafts, or whether recruitment of CyaA into rafts precedes calcium influx and AC translocation.

We showed here that calpeptin-mediated inhibition of calcium-activated processing of talin by calpain yields (i) inhibition of CyaA recruitment into rafts and (ii) it inhibits AC translocation across membrane. Collectively, hence, these results strongly suggest that the transient influx of Ca^2+^ into cells accompanies the earliest step of membrane insertion of the toxin ‘translocation intermediate’. This would precede and be essential for subsequent recruitment of CyaA into lipid rafts, whereupon AC translocation is accomplished.

It remains, however, to be determined what is the threshold of the calcium signal required for initiation of talin cleavage and mobilization of CyaA into lipid rafts. Two major isoforms of calpain have, indeed, been so far identified in eukaryotic cells. The calpain I (μ-calpain) is activated at µM Ca^2+^ concentrations, while calpain II (m-calpain) only responds to mM concentrations of Ca^2+^ [57]. Here we observed that CyaA relocalization into DRM occurred at 1 nM toxin concentration, which is about two-times less than the lowest CyaA concentrations still allowing to elicit a [Ca^2+^]*_i_* increase detectable in cells by the Fura-2/AM probe [18]. Moreover, only influx of extracellular Ca^2+^ ions into cells, and not the elevation of cytosolic [Ca^2+^]*_i_* due to Ca^2+^ release from intracellular stores, enabled the accumulation of CyaA in DRM (cf. Fig. 4). This differs importantly from the mechanism reported for localization of the leukotoxin (LtxA) of *Actinobacillus actinomycetemcomitans* into rafts. LtxA binds yet another β_2_ integrin of human leukocytes, the LFA-1 or CD11a/CD18 heterodimer. Horeover, LtxA appears to first adsorb on cell membrane of T lymphocytes in a receptor-independent manner, to trigger, somehow the store-operated elevation of cytosolic [Ca^2+^]*_i_*, to induce talin cleavage, and upon relocation into rafts, the Ltx clusters with LFA-1 within rafts to promote cell lysis [58].

With CyaA, all the Ca^2+^ ions entering macrophage cytoplasm due to toxin action appear to come from extracellular medium [18]. It is generally accepted that there exists a gradient of about four orders of magnitude in Ca^2+^ concentrations between the external medium (∼2 mM) and cell cytosol (∼100 nM). Therefore, numerous Ca^2+^-buffering proteins accumulate beneath the inner face of cell membrane, accounting for formation of local Ca^2+^ gradients and controlling signaling induced by alterations of Ca^2+^ concentrations in the submembrane compartment. These concentrations can, indeed, be still much higher, and rise more rapidly, than the bulk Ca^2+^ levels in cell cytosol [59]. Therefore, it is likely that even an importantly lower CyaA concentration than used here (1 nM = 176 ng/ml), may still be generating sufficiently high local Ca^2+^ signal beneath cell membrane in order to promote activation of μ-calpain at the inner face of cell membrane. It appears, thus, plausible to assume that mobilization of CyaA into rafts in phagocyte membrane, and translocation of the AC domain from rafts directly into the cytosolic compartment of phagocytes, are indeed taking place also during natural *Bordetella* infections *in vivo*. This would account for the remarkable efficacy of CyaA in disarming the sentinel cells of the host innate defense.

## Materials and Methods

### Expression and purification of CyaA-derived proteins

Intact recombinant CyaA and its mutant variants were expressed and purified as previously described [19]. Except of pro-CyaA, the CyaA proteins were produced in *E. coli* XL1-Blue in the presence of the co-expressed toxin-activating acyltransferase CyaC, as previously described [20]. Lipopolysaccharide was eliminated by repeated 60% isoporopanol washes of CyaA bound to the Phenyl Sepharose resin [60]. This reduced the final endotoxin content below 50 EU/mg of purified protein, as determined by the *Limulus* amebocyte lyzate assay (QCL-1000, Cambrex, NJ, USA). For fluorescence microscopy, the CyaA proteins were labeled while bound to Phenyl-Sepharose resin during the final purification step. Briefly, the CyaA eluates from a DEAE-Sepharose columns (GE Healthcare) in 50 mM Tris-HCl (pH 8), 8 M urea, 0.2 mM CaCl_2_, 200 mM NaCl, were diluted 1∶4 with a buffer containing 50 mM Tris-HCl (pH 8), 1 M NaCl and 1 mg of CyaA was loaded on an 0.5 ml Phenyl-Sepharose column. The columns were extensively washed with 0.1 M sodium bicarbonate (pH 9), 1 M NaCl. Next 10 µg/ml Alexa Fluor 488 succinimidylester solution (Molecular Probes) was loaded and labeling proceeded at 25°C for 1 hour. The columns were washed with 50 mM Tris-HCl (pH 8), 1 M NaCl, and the CyaA-Alexa Fluor 488 conjugates were eluted in a buffer containing 50 mM Tris-HCl (pH 8), 8 M urea and 2 mM EDTA. Unreacted dye was separated from labeled CyaA on Sephadex G-25 columns (GE Healthcare). Efficiency of protein labeling was assessed spectrophotometrically and a molar ratio of about 1∶4 (protein∶dye) was found for all CyaA preparations. It was verified that this extent of labeling did not affect the biological activities of CyaA.

### Antibodies, reagents, cell lines, SDS-PAGE, BN-PAGE and Western blotting

See Protocol S1 for full description.

### Isolation of detergent-resistant membranes

Detergent-resistant membranes (DRM) were separated by flotation in discontinuous sucrose density gradients. Briefly, J774A.1 cells (2.10^7^) were washed with prewarmed DMEM and incubated with 1 nM CyaA proteins at 37°C for 10 min. Cells were washed with ice-cold phosphate-buffered saline (PBS), scraped from the Petri dish and extracted at 4°C for 60 min using 200 µl of TBS buffer (20 mM Tris-HCl, pH 7.5, 150 mM NaCl) containing 1% Triton X-100, 1 mM EDTA, 10 mM NaF and a Complete Mini proteinase inhibitor cocktail (Roche, Basel, Switzerland). The lyzates were clarified by centrifugation at 250×*g* for 5 min and the post-nuclear supernatants were mixed with equal volumes of 90% sucrose in TBS. The suspensions were placed at the bottom of centrifuge tubes and overlaid with 2.5 ml of 30% sucrose and 1.5 ml of 5% sucrose in TBS. Membrane flotation according buoyant density was achieved by centrifugation at 150,000×*g* in a Beckman SW60Ti rotor for 16 h at 4°C. Fractions of 0.5 ml were removed from the top of the gradient.

### Ca^2+^ influx into cells

Calcium influx into J774A.1 and U937 cells was measured as previously described [18]. Briefly, cells were loaded with 3 µM Fura-2/AM (Molecular Probes) at 25°C for 30 min and the time course of calcium entry into cells induced by addition 3 µg/ml of CyaA proteins was determined as ratio of fluorescence intensities (excitation at 340/380 nm, emmision 505 nm), using a FluoroMax-3 spectrofluorometer equipped with DataMax software (Jobin Yvon Horriba, France).

### Depletion of cholesterol

J774A.1 cells were incubated in DMEM supplemented with 10 mM methyl-β-cyclodextrin (MβCD) at 37°C for 30 min. Cholesterol-depleted U937 cells were obtained upon growth in RPMI medium supplemented with 10% of delipidated serum (lipoprotein-deficient serum from fetal calf, Sigma) for 48 h. Cholesterol content was determined using an Amplex Red Cholesterol Assay Kit (Molecular Probes, Invitrogen) according to manufacturer's instructions. Viability of cells was tested by trypan blue staining and no significant cell death occurred upon cholesterol extraction.

### Fluorescence microscopy

U937 cells were grown in media with 10% standard, or delipidated serum, and were mounted on polylysin-coated coverslips prior to incubation with labeled proteins. J774.A1 cells (5.10^4^) were grown directly on coverslips (∅ φ12 mm) and incubated with Alexa Fluor 488-labeled CyaA proteins (6 nM) at 37°C for 10 min, before cells were washed and 5 µg/ml of Alexa Fluor 594-labeled cholera toxin subunit B (CtxB) was added for additional 5 min. The unbound proteins were washed-off with ice-cold PBS, cells were fixed with 4% paraformaldehyde in PBS at 25°C for 20 min, and mounted in Mowiol solution (Sigma). Fluorescence images were taken using a Cell^R^ Imaging Station (Olympus, Hamburg, Germany) based on Olympus IX 81 fluorescence microscope, using a 100× oil immersion objective (N.A. 1.3). Digital images were processed using ImageJ software.

### Immunoprecipitation

J774.A1 cells (10^6^) were incubated with 17 nM CyaA in DMEM for 30 min at 37°C, washed with Hank's Buffered Salt Solution buffer (HBSS), and lyzed at 4°C during 30 min in 500 µl of Tris-buffered saline (pH 7.4) supplemented with 1% Triton X-100 and EDTA-free Complete Mini proteinase inhibitor cocktail (Roche, Basel, Switzerland). The lyzate was centrifuged for 15 min at 10,000×*g* at 4°C, and the supernatant was incubated with MEM-174 MAb covalently linked to CNBr-activated Sepharose beads (GE Healthcare) at 4°C for 1 h. The beads were washed five times with 1 ml of the lysis buffer and the bound proteins were eluted with SDS-PAGE loading buffer and analyzed by SDS-PAGE followed by Western blotting.

### CyaA binding to cells

J774A.1 or U937 cells (10^6^) were incubated with 6 nM CyaA proteins at 37°C for 10 min, washed repeatedly in buffer, and the amount of cell-associated adenylate cyclase (AC) activity was determined in cell lyzates as previously described by [61].

### cAMP assay

J774A.1 or U937 cells (10^6^) were incubated with CyaA proteins at indicated concentrations for 10 min at 37°C and intracelular cAMP concentrations were determined in cell lyzates using a competitive ELISA as previously described [62].

## Supporting Information

Figure S1 Cholesterol depletion does not affect the tight binding of CyaA to CD11b/CD18. J774A.1 (10^5^) cells were pretreated with 10 mM MβCD at 37°C for 30 min and placed on ice before CyaA or pro-CyaA were added at indicated concentrations. Proteins were allowed to bind CD11b/CD18 for 30 min at 4°C, before 30 nM biotinylated CyaA was added for another 30 min on ice. Cells were washed, stained with phycoerythrin-streptavidin conjugate and amounts of bound biotinylated CyaA were determined by flow cytometry. Results are expressed as relative binding of biotinylated CyaA according to the formula = (sample binding)/(maximum binding)×100. Data shown are the mean ± S.D. from three independent experiments performed in duplicates.
(0.16 MB DOC)

Protocol S1 Supplementary methods.
(0.03 MB DOC)
