# Supplementary material for: Bordetella Adenylate Cyclase Toxin Mobilizes Its β2 Integrin Receptor into Lipid Rafts to Accomplish Translocation across Target Cell Membrane in Two Steps
Source: PLoS Pathog. 2010 May 13;6(5):e1000901. doi: 10.1371/journal.ppat.1000901 (PMC2869314; doi:10.1371/journal.ppat.1000901)
Supplement: Figure S1 — Cholesterol depletion does not affect the tight binding of CyaA to CD11b/CD18. J774A.1 (105) cells were pretreated with 10 mM MβCD at 37°C for 30 min and placed on ice before CyaA or pro-CyaA were added at indicated concentrations. Proteins were allowed to bind CD11b/CD18 for 30 min at 4°C, before 30 nM biotinylated CyaA was added for another 30 min on ice. Cells were washed, stained with phycoerythrin-streptavidin conjugate and amounts of bound biotinylated CyaA were determined by flow cytometry. Results are expressed as relative binding of biotinylated CyaA according to the formula = (sample binding)/(maximum binding)×100. Data shown are the mean ± S.D. from three independent experiments performed in duplicates. (0.16 MB DOC) [file ppat.1000901.s001.doc]

**Supplementary Figure S1**

**
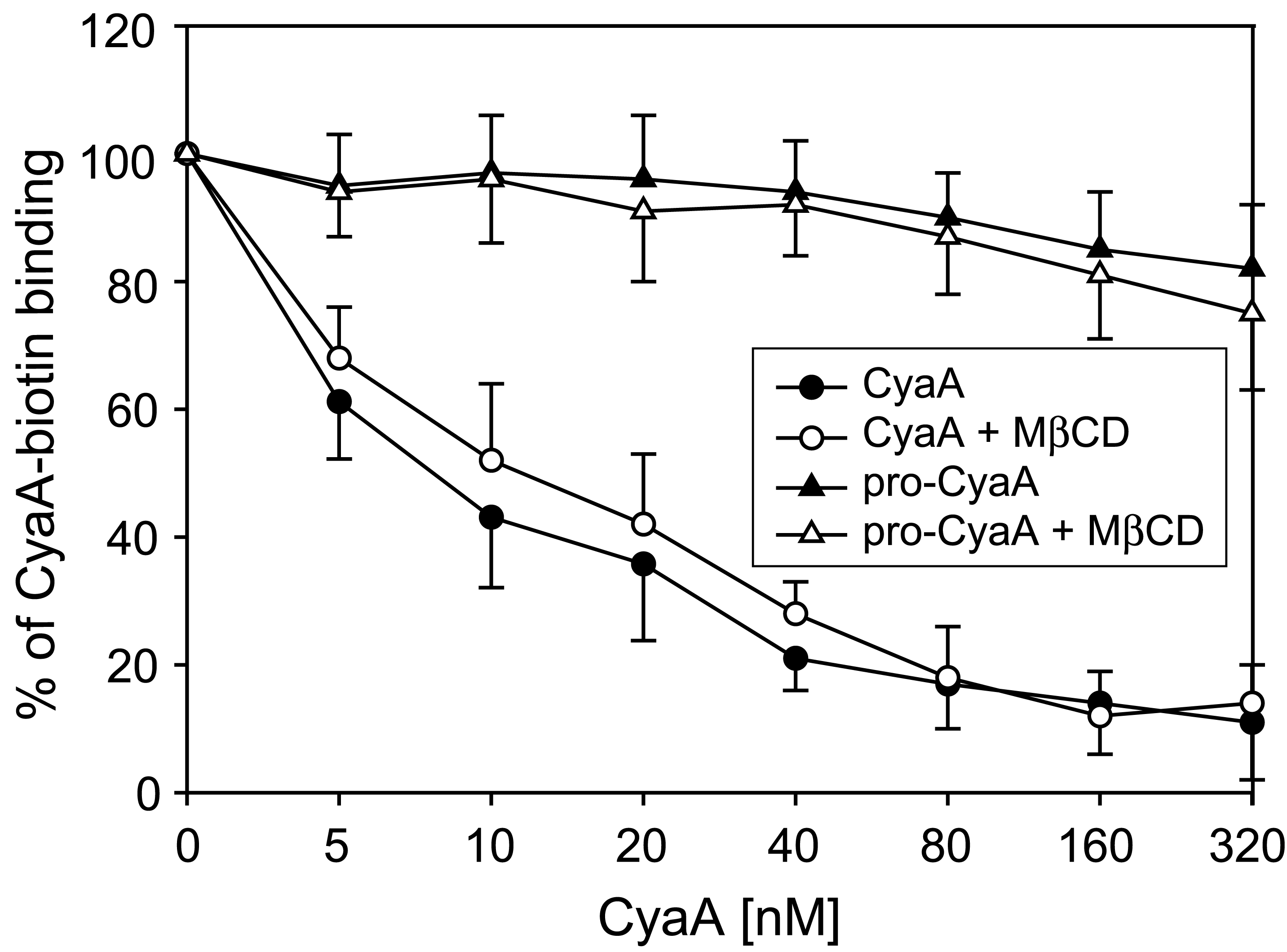
**

**Fig. S1. Cholesterol depletion does not affect the tight binding of CyaA to CD11b/CD18.** J774A.1 (10^5^) cells were pretreated with 10 mM MβCD at 37^o^C for 30 min and placed on ice before CyaA or pro-CyaA were added at indicated concentrations. Proteins were allowed to bind CD11b/CD18 for 30 min at 4^o^C, before 30 nM biotinylated CyaA was added for another 30 min on ice. Cells were washed, stained with phycoerythrin-streptavidin conjugate and amounts of bound biotinylated CyaA were determined by flow cytometry. Results are expressed as relative binding of biotinylated CyaA according to the formula = (sample binding)/(maximum binding)×100. Data shown are the mean ± S.D. from three independent experiments performed in duplicates.
