# Supplementary material for: Bordetella Adenylate Cyclase Toxin Mobilizes Its β2 Integrin Receptor into Lipid Rafts to Accomplish Translocation across Target Cell Membrane in Two Steps
Source: PLoS Pathog. 2010 May 13;6(5):e1000901. doi: 10.1371/journal.ppat.1000901 (PMC2869314; doi:10.1371/journal.ppat.1000901)
Supplement: Protocol S1 — Supplementary methods. (0.03 MB DOC) [file ppat.1000901.s002.doc]

**Supplementary Methods**

**Antibodies and reagents**

The antibodies used in this study were mouse anti-talin (8d4; Sigma), anti-talin head (TA-205; AbDSerotec, Oxford, UK), anti-α-tubulin (Tu-01; Exbio, Prague, Czech Republic), anti-CD71 (MEM-189; Exbio), anti-CD11a (MEM-25; Exbio). Mouse monoclonal anti-CyaA antibodies (9D4 and 3D1) were kindly provided by Erik L. Hewlett (University of Virginia School of Medicine, Charlottesville, USA). Mouse anti-NTAL (NAP-08) was a gift of Pavla Angelisova (Institute of Molecular Genetics, Prague, Czech Republic). The hybridoma cell line producing mouse anti-CD11b antibody (clone OKM-1) was obtained from American Type Culture Collection (ATCC CRL-8026) and the MAb was purified from culture supernatant using recombinant protein G column chromatography. Amplex Red Cholesterol Assay Kit was purchased from Molecular Probes (Invitrogen, Carlsbad, USA). Calpeptin was obtained from Calbiochem (Merck, Darmstadt, Germany) and methyl-β-cyclodextrin (MβCD) was from Sigma (St. Louis, USA).

**Cell lines and growth conditions**

The cell lines used in the experiments were J774A.1 mouse macrophages (ATCC TIB 67), U937 human leukemic monocyte lymphoma (ATCC **CRL-1593.2**), CHO-K1 Chinese Hamster Ovary cell line (ATCC CCL 61). The CHO-CD11b/CD18 cells were CHO-K1 cells transfected with genes for human CD11b/CD18 (a kind gift of D. Golenbock, Boston University School of Medcine, Boston, MA, USA). The J774A.1 and U937 cells were maintained at 37°C under 5% CO_2_ in RPMI medium 1640 containing 10% (v/v) heat-inactivated fetal calf serum (FCS, Biochrom AG, Berlin, Germany) and antibiotic-antimycotic solution (0.1 mg/ml streptomycin, 1000 units/ml penicillin, and 0.25 µg/ml amphotericin; Sigma). The CHO-K1 and CHO-CD11b/CD18 cells were grown at 37°C under 5% CO_2_ in Ham’s F12 medium (Gibco, Invitrogen) supplemented with 10% (v/v) heat-inactivated FCS and antibiotic antimycotic solution.

**SDS-PAGE, BN-PAGE and Western blotting**

SDS-polyacrylamide gel electrophoresis (SDS-PAGE) was performed according to standard protocols. Samples for Blue Native-Polyacrylamide Gel Electrophoresis (BN-PAGE) were solubilized with *n*-Octyl-*β*-D-glucopyranoside (30 mM final concentration) for 30 min on ice, mixed with a Blue native loading buffer consisting of 50 mM Bis-tris, pH 7.0, 750 mM aminocaproic acid, and 0.25% Coomassie Brilliant Blue G, and analyzed on 5% non-denaturing gel in a Tricine buffer. For Western blotting, separated proteins were transferred to nitrocellulose membrane (Immobilon-P, GE Healthcare). Membranes were blocked with 5% nonfat milk in Tris-buffered saline containing 0.05% Tween-20 (TBST) for 1 h at room temperature, incubated with primary antibodies in TBST containing 5% bovine serum albumin overnight at 4 °C prior to incubation with horseradish peroxidase-conjugated goat anti-mouse antibody (GE Healthcare) diluted in 5% nonfat milk TBST for 1 h at room temperature. Detection was carried out using a SuperSignal West Femto Maximum Sensitivity Substrate chemiluminescence reagent kit (Pierce, Thermo Fisher Scientific).

**Competitive CyaA binding**

J774A.1 (2.10^5^) cells were preincubated with indicated concentrations of CyaA proteins in DMEM for 30 min on ice before CyaA-biotin was added to a 30 nM concentration. After washing the cells were stained with streptavidin-PE (Pharmingen) and analyzed by flow cytometry using a BD LSR II instrument (BD Biosciences). Aggregated and dead cells were subtracted by gating for Hoechst 33258 exclusion. Binding data were deduced from the mean fluorescence intensity (MFI) and expressed as percentage of binding = (sample binding) / (maximum binding) × 100. The maximum binding corresponds to (MFI value of cells incubated with CyaA in the absence of CyaA-biotin competitor) - (MFI value of cells incubated with medium alone). The sample binding corresponds to (MFI value of cells incubated with CyaA in the presence of competitor) - (MFI value of cells incubated with medium alone).
